# Using Fish Population Metrics to Compare the Effects of Artificial Reef Density

**DOI:** 10.1371/journal.pone.0139444

**Published:** 2015-09-30

**Authors:** Catheline Y. M. Froehlich, Richard J. Kline

**Affiliations:** Department of Biological Sciences, University of Texas at Brownsville, Brownsville, Texas, United States of America; North Carolina State University, UNITED STATES

## Abstract

Artificial reefs continue to be added as habitat throughout the world, yet questions remain about how reef design affects fish diversity and abundance. In the present study, the effects of reef density were assessed for fish communities and sizes of economically valuable *Lutjanus campechanus* 13 km off Port Mansfield, Texas, at a reef composed of more than 4000 concrete culverts. The study spanned from May to June in 2013 and 2014, and sites sampled included natural reefs, bare areas, and varying culvert patch density categories, ranging from 1–190 culverts. Abundances of adults and species evenness of juvenile populations differed between the years. Fish communities did not significantly differ among density categories; however, highest species richness and total abundances were observed at intermediate culvert densities and at natural reefs. Whereas the abundance of *L*. *campechanus* did not differ among density categories, mean total lengths of *L*. *campechanus* were greatest at the lower density. Our findings suggest that reefs should be deployed with intermediate patch density of 71–120 culverts in a 30-m radius to yield the highest fish abundances.

## Introduction

Natural reef cover is sparse or diminishing in many locations, including the Middle East, Pacific Ocean, Caribbean Sea, and the Gulf of Mexico [1–4]. Artificial reef deployment continues to be a regular response to the limited natural habitat in the interest of sustaining reef fish populations, tourism, and commercial fishing [3]. Despite the widespread use of artificial reefs, there remains a need to understand key factors influencing fish diversity and abundance.

Concrete is commonly used for artificial reefs due to its availability, low cost, nontoxic characteristics, and durability [5]. Concrete reefs have been documented to hold high fish diversity [6], and have been arranged into different patterns, or simply deployed at random in an area [5,7]. Such arrangements can alter habitat complexity, which has been found to affect reef communities [8–10]. Some studies note that higher reef complexity increases species richness and abundance, and reduces mortality, yet the type of complexity reported (rugosity, total volume, number of refugia, or vertical structure) varies widely [10–16]. Thus, determining the ideal habitat complexity of concrete structure, in order to maximize fish species richness and abundance, could result in increased value of future deployments.

Artificial reefs have been attributed to improved sport and commercial fisheries [3,17]. Anglers typically desire larger fishes, thus identifying ideal habitat for economically valuable fishes, such as groupers and snappers, is important for fisheries management. More information is needed because of current debates regarding the role of artificial reefs in the life cycle of *Lutjanus campechanus* and its over-fished status in the Gulf of Mexico (GOM) since the 1980s [18–20]. *Lutjanus campechanus* is a relatively long-lived fish commonly found over hard structure in the GOM that undergoes ontogenic habitat shifts as it grows [3,21,22]. Early in life, *L*. *campechanus* utilize low-relief, rubble-shell habitat, then move to more complex habitats, such as nearshore reefs [3]. Years later, individuals may move to deeper reefs, such as oil platforms, rock outcroppings and larger artificial reefs [3,23]. These ontogenic shifts suggest that artificial reef complexity and size may be important factors influencing the age and size of *L*. *campechanus* over different reefs.

In research comparing artificial reefs to local natural reefs, some studies have found that natural reefs have higher species abundance and richness [24–26]. More artificial concrete reef placements are predicted in the near future in the GOM, and assessing fish communities on concrete reefs could provide important information to guide future deployments. To address the lack of data on habitat complexity and the need for local population analyses of economically valuable fishes, surveys were conducted off the coast of Texas, in the northwestern Gulf of Mexico. The present study assessed fish diversity and community differences between two consecutive years among varying categories of concrete reef patch density and nearby natural reefs. In addition, *L*. *campechanus* populations and mean total lengths were compared separately across all reef categories. Our predictions were that:

1. Patch reefs with higher concrete density would have higher fish diversity and abundance.
2. Natural reef patches would have the highest diversity of fishes and abundance of juveniles due to their structural complexity, as compared to concrete reef patches.
3. *Lutjanus campechanus* would be largest at the densest concrete reefs, but smallest at natural reefs because of their low-relief, which is characteristic of juvenile habitat.

## Materials and Methods

### Site Description

The study was conducted at the PS-1047 Reef (N 26° 31.535’–W 97° 09.215’), which has an average depth of 21 m. PS-1047 is part of the Texas Artificial Reef Nearshore Reefing Program, and is located 13 km from the Port Mansfield jetties in Texas state waters. As of August 21, 2011, 4,922 concrete culverts of various sizes occupied the sand and mud sea floor in patch formations. Natural reef patches were also identified at the fringes of the reefing area and outside of the reefing area at the same 21-m depth. Natural reefs were composed of hard clay and sandstone base with solitary soft corals and octocorals, algae, sponges, and polychaete worms. The natural reef patches surveyed in this study were similar to Seven and One-Half Fathom Reef, a well-studied reef of lacustrine origin composed of hard clay and sandstone with sponges, hydroids, ascidians, and polychaete worms [27–31].

### Characterization and Selection of Reef Categories

**Fig 1:**
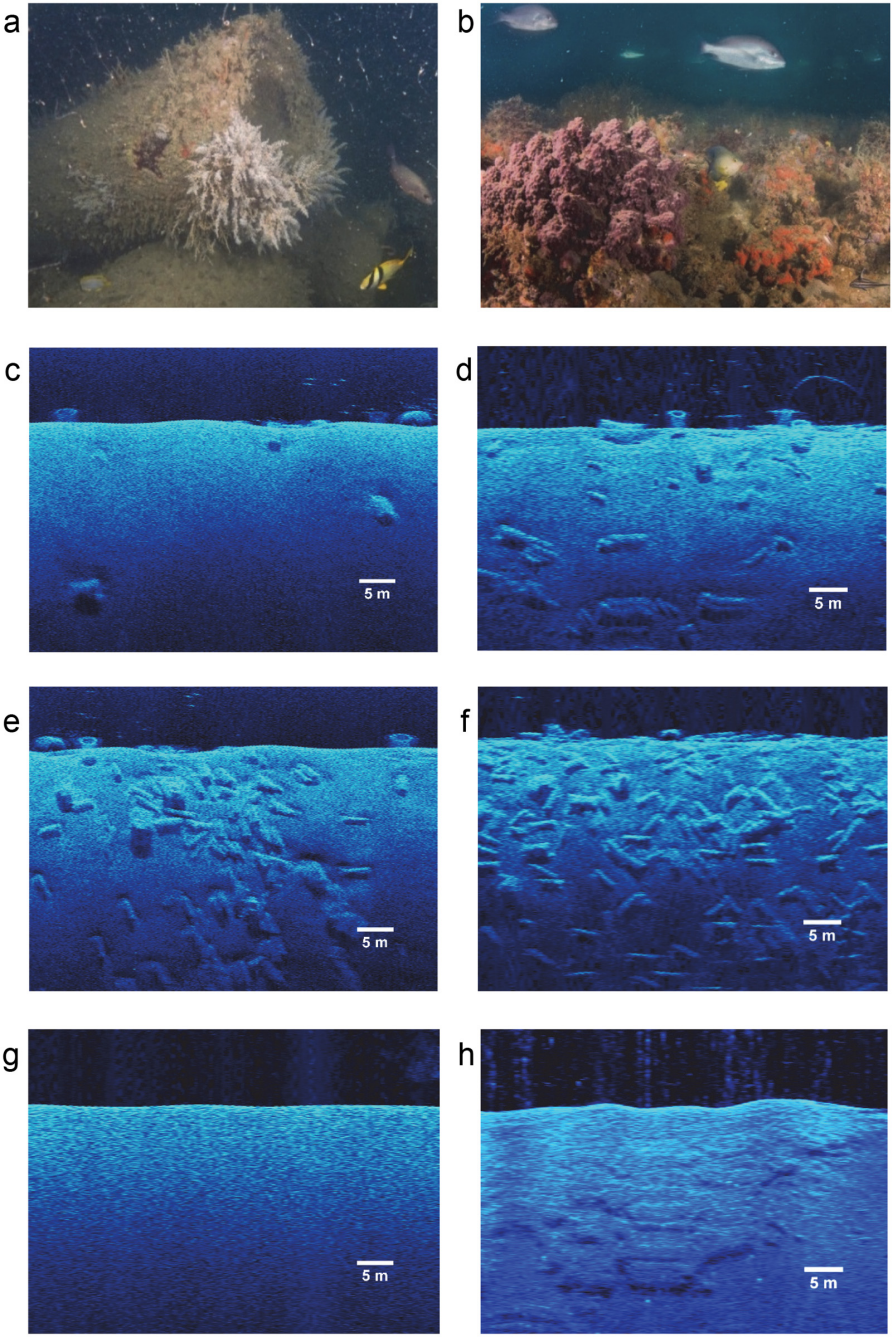
(a) Culvert reef and (b) and naturally-occurring reef sites were analyzed in different categories: (c) CC1: 1–30 culverts, (d) CC2: 31–70 culverts, (e) CC3: 71–120 culverts, (f) CC4: 121–190 culverts, (g) bare areas, and (h) natural reefs. Images (c) to (h) were produced by ImageJ software.

At each survey site, all structure within a 30-m radius was quantified, and six patch categories were used overall (Fig 1): natural reefs (N), bare areas with no hard structure (B), and four culvert categories: CC1 (1–30 culverts), CC2 (31–70 culverts), CC3 (71–120 culverts), and CC4 (121–190 culverts). There was at least 100 m of bare sand between sites to reduce the chances of encountering the same fish.

Initially, culvert sites were located with a low resolution survey map provided by Texas Parks and Wildlife Department. Each potential site was further surveyed by SCUBA divers to assess the type of structure present. Later, a side scan sonar unit (Humminbird 1198s SI @ 455 kHz, Johnson Outdoors Marine Electronics, Inc., Eufaula, Alabama, and towfish, The Tank, First Response Outfitters, Houston, Texas) was acquired to further characterize existing sites, and locate additional sites, including natural patches. In June and July of 2013, twelve sites consisting of four categories (sample size) were sampled: B (3); CC1 (3); CC2 (3); & CC4 (3). The same sites were sampled again in May to July 2014. Additionally in 2014, more reef sites were surveyed for a total of 20 sites and five categories consisting of: CC1 (4); CC2 (3); CC3 (3); CC4 (4); and N (6).

To characterize each survey site, still frames from three 200-m transect lines of side scan sonar (40.176 pixels/m) were produced from SonarTRX software (Leraand Engineering, Inc., version 13.1, Honolulu, Hawaii). The frames were later imported into ImageJ processing software (National Institute of Health, version 1.48, Bethesda, Maryland) to count culverts, and to better describe the culvert categories. While the side scan sonar in this study was used to detect large scale differences in relief (>0.2 m), small complexity differences (<0.1 m) were not measured. Additional measures of habitat characteristics were calculated from ImageJ, including substrate rugosity, percent substrate cover (%), and vertical relief (height, m). To measure substrate rugosity, a line was drawn tracing the structural relief over a 60-m length of the reef site. The length of the line was then divided by 60 m to calculate a substrate rugosity index for each site. Percent substrate cover was calculated by measuring how much of the reef site (in a 30-m radius circle) was covered by structure. Average and maximum vertical relief were calculated by measuring substrate height every meter along the 60-m length of the reef site.

### Census of Reef Fishes

Visual SCUBA fish surveys were conducted for 15 min as follows: reef-associated fishes were quantified via stationary cylinder census [32], and cryptic species and juveniles were quantified with a roving survey around the structures. Adult and juvenile fishes were categorized separately in the field, based on coloration and body size. Horizontal reef visibility over the reef was determined with a Secchi disk (United Scientific Inc., Little Canada, Minnesota) and a tape measure.

### Measurements of *Lutjanus campechanus*

Alongside the surveys in 2014, a second diver recorded *L*. *campechanus* lengths using parallel lasers, 0.3 m apart, mounted on an aluminum flat bar (3.9 x 70.0 x 0.3 cm) with a high definition camera attached (GoPro HD Hero 3+, GoPro Inc., San Mateo, California). The second diver aimed the lasers at the broad side of *L*. *campechanus* to record as many as possible in the 15-min period. To reduce the chances of sampling the same individual, the diver stayed at the same location in the water column. *Lutjanus campechanus* total lengths were collected from scaled videos and manual measurements using a ruler in Adobe Premier Pro software (Adobe Inc., version 7.2, San Jose, California). In some cases, video attenuation did not allow for accurate estimation of *L*. *campechanus* sizes, and these recordings were excluded. On average, 78 ± 8 *L*. *campechanus* were measured in each reef category. Ages were then estimated from 558 total length measurements using the Von Bertalanffy Growth Model derived by Syc (2011):

$${\text{TL}}_{\text{t}}\;=\;936.37\;(1\text{- }{\text{e}}^{\left(-0.205\;(\text{t}+0.142)\right)}\text{)}$$

where TL was the total length of fish, and t was the age at total length.

### Statistical Analyses

For adult, juvenile, and whole community abundances, the following analyses were applied. Species abundances in sites sampled both years were square-root transformed, and were compared against initial reef categories and year in a two-way permutational analysis of variance (PERMANOVA), using the Bray-Curtis similarity index [33]. Reef visibility was assessed as a covariate. To assess significant clustering of samples based solely on species abundances, but no other factors, Hierarchical Cluster (CLUSTER) analysis and the Similarity Profiles (SIMPROF) routine analysis were conducted. The Similarity Percentages (SIMPER) routine was run to determine what species contributed to similarities and dissimilarities among different sites, reef categories, and years.

Similar analyses were conducted to assess species abundances of adults, juveniles, and whole communities over all reef categories surveyed in 2014 in one-way PERMANOVAs. Principal Components Analysis (PCA) was conducted to assess how sample sites differed among substrate rugosity, average vertical relief, maximum vertical relief, and percent cover. All multivariate analyses were performed using PRIMER-E v6 package.

Species indices (species richness, evenness, diversity, and total abundance) were assessed over reef categories and year using two-way analyses of variance (ANOVA). Species indices from 2014, with five reef categories were assessed using one-way ANOVAs. *Lutjanus campechanus* abundances and lengths were both compared among reef categories in 2014 using one-way ANOVAs. All univariate analyses were performed using SPSS IBM Statistics v20 package.

### Ethics Statement

No specific permissions were required for any of the activities undertaken during the research at any of the sites visited. The study was conducted at a publicly accessible reef (PS-1047) that is in Texas state waters, and is part of the Texas Artificial Reef Nearshore Reefing Program. The reef does not lie in protected areas, or in a state park or national park. Endangered or protected species were not involved during any part of the field studies. Vertebrates were studied in the water via visual SCUBA surveys and via video recordings. No vertebrates were collected or sacrificed, and no contact with animals was conducted during any part of the field studies. No approval was required from the Institutional Animal Care and Use Committee, and no field permits were necessary during any part of the experiment.

## Results

### Fish Communities over Two Consecutive Years

Throughout the course of the study, there were 59 species censused across 27 families (S1 Appendix). The most abundant species were *Balistes capriscus*, *Haemulon aurolineatum*, *Lutjanus campechanus*, *Parablennius marmoreus*, *Pareques umbrosus*, and *Serranus subligarius*. Bare areas did not exhibit reef-associated fishes. Among culvert categories, no significant differences were found among overall community compositions (PERMANOVA: pseudo-F(1,3,19) = 0.844, P = 0.622) or between years (pseudo-F(1,3,19) = 1.196, P = 0.271), using multivariate approaches. Reef visibility did not predict differences between surveys regardless of the year (pseudo-F(1,2,18) = 0.951, P = 0.500). However, higher adult abundances were observed in 2013 than in 2014 (Table 1, ANOVA: F(1,2,12) = 6.191, P = 0.029). SIMPER revealed that fewer *Caranx crysos*, *Chaetodipterus faber*, and *Lutjanus griseus* adults were observed in 2014. Juveniles had higher species evenness in 2013 than in 2014 (Table 1, ANOVA, F(1,2,12) = 6.584, P = 0.025). SIMPER revealed that *Haemulon aurolineatum*, *Halichoeres bivittatus* and *P*. *umbrosus* juveniles were generally observed in higher abundances in 2014 than in 2013, and *Seriola dumerili* were only seen in 2014. On the other hand, slightly more *Stegastes variabilis* juveniles were censused in 2013.

**Table 1:** Species indices of culvert reefs censused from May to July in 2013 and 2014.

	Species Index	All Individuals	Juveniles	Adults
**2013**	Species Richness	13 ± 4	2 ± 0.2	11 ± 2
	Total Abundance	142 ± 68	17 ± 11	125 ± 26 *
	Species Evenness	0.65 ± 0.14	0.72 ± 0.09 *	0.67 ± 0.04
	Shannon-Weaver Index	1.66 ± 0.45	0.61 ± 0.10	1.55 ± 0.13
**2014**	Species Richness	11 ± 1	3 ± 1	9 ± 1
	Total Abundance	121 ± 27	62 ± 24	59 ± 10
	Species Evenness	0.65 ± 0.02	0.33 ± 0.12	0.68 ± 0.03
	Shannon-Weaver Index	1.54 ± 0.10	0.51 ± 0.16	1.43 ± 0.12
**Total**	Species Richness	12 ± 1	3 ± 0.4	9 ± 1
	Total Abundance	131 ± 17	39 ± 14	92 ± 16
	Species Evenness	0.65 ± 0.03	0.52 ± 0.09	0.67 ± 0.02
	Shannon-Weaver Index	1.60 ± 0.09	0.56 ± 0.09	1.49 ± 0.09

* Significant differences (P < 0.05) between the two years

### Fish Communities Over Varying Reef Categories in 2014

**Fig 2:**
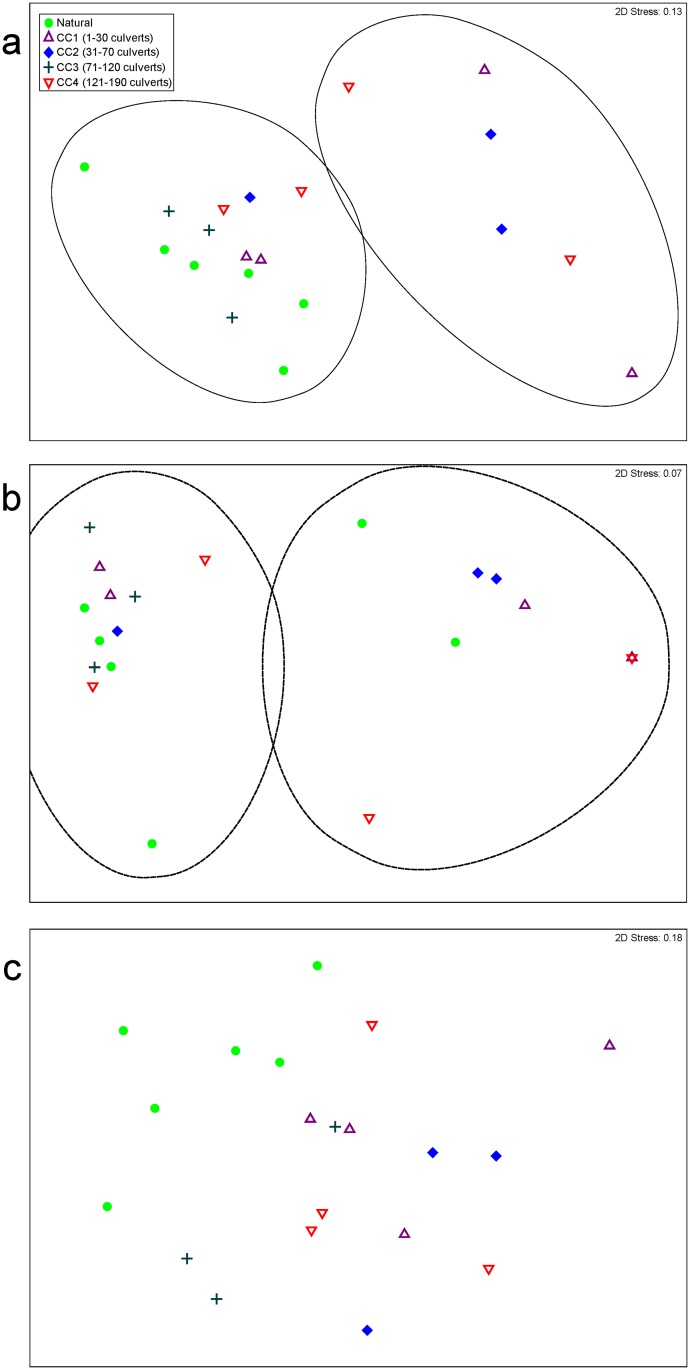
Community composition of (a) all fishes, (b) juvenile fishes, and (c) adult fishes in varying density categories of culvert and natural reefs in 2014. Solid and dashed ovals indicate an overlay of Hierarchical Cluster Analysis that defined two clusters of samples as being significantly different from one another. Clusters in solid ovals have at least 44% similar communities, and clusters in dashed ovals have at least 20.3% similarity.

**Fig 3:**
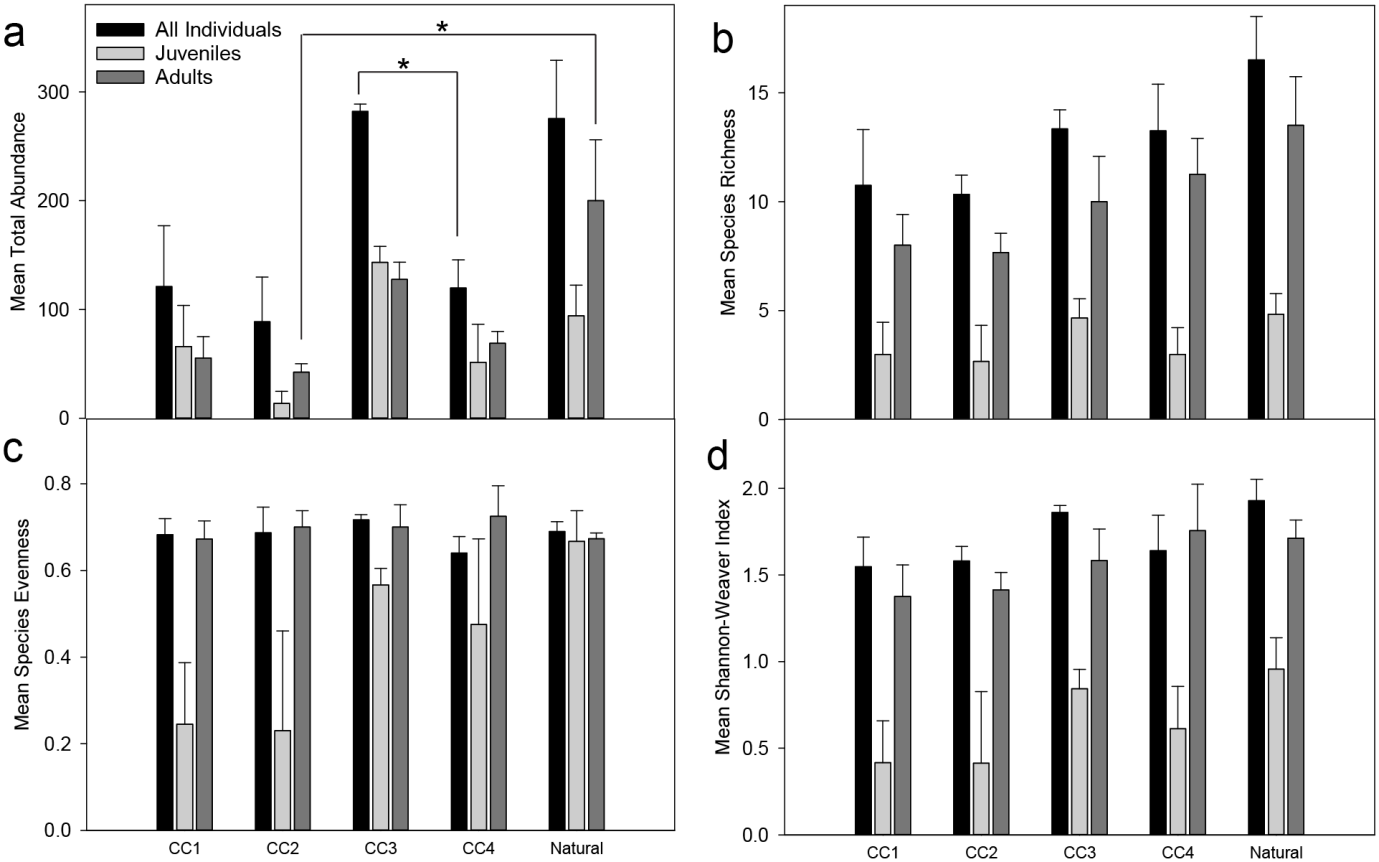
(a) Total abundances, (b) species richness, (c) species evenness, and (d) Shannon-Weaver indices of fish communities in varying reef categories in 2014. CC1: 1–30 culverts, CC2: 31–70 culverts, CC3: 71–120 culverts, CC4: 121–190 culverts, and Natural: naturally-occurring reefs. Error bars represent standard error (± 1). Asterisks (*) indicate significant differences (P > 0.05).

In 2014, *B*. *capriscus*, *Haemulon aurolineatum*, *Halichoeres bivittatus*, *L*. *campechanus*, *P*. *marmoreus*, and *S*. *subligarius* were the most abundant species censused. Fish community compositions, including all adults and all juveniles, did not significantly differ among reef categories in 2014 (Fig 2a, pseudo-F(4,15) = 1.516, P = 0.076), in multivariate analyses. Total abundance of all fishes significantly varied among reef categories (Welch statistic = 11.899, DF1 = 4, DF2 = 6.166, P = 0.005). Significantly higher total abundances of fishes were observed at CC3, 282 ± 7 individuals, than at CC4, 120 ± 26 individuals (Fig 3a, Games-Howell: P = 0.026). Other species indices were not significantly different among reef categories. However, category CC3 and natural sites consistently had the highest observed values of total abundance, species evenness, and diversity (Fig 3). CLUSTER analysis produced two significant clusters of samples (Fig 2a, SIMPROF: π = 4.18, P = 0.010). Cluster-1 contained all natural and CC3 sites in addition to some sites for CC1, CC2 and CC4. Cluster-2 included the remaining sites from CC1, CC2 and CC4 categories. SIMPER indicated that *B*. *capriscus*, *E*. *adscensionis*, *Haemulon aurolineatum*, *Halichoeres bivittatus*, *L*. *campechanus*, *L*. *griseus*, *Parablennius marmoreus*, *Pareques umbrosus*, *Seriola dumerili*, and *Stegastes variabilis* exhibited higher abundances in Cluster-1. *Epinephelus adscensionis* was only observed in Cluster-1, whereas *Archosargus probatocephalus* was observed in higher abundances in Cluster-2.

Juvenile community compositions did not significantly differ among reef categories (Fig 2b, pseudo-F(4,15) = 1.072, P = 0.391). No significant differences were seen among juvenile species indices and reef categories, but CC3 and natural sites had the highest observed values for juvenile species richness, diversity, and total abundance (Fig 3). CLUSTER analysis revealed two significant clusters with samples that were at least 20.3% similar (Fig 2b, π = 6.88, P = 0.010). The two clusters exhibit similar site groupings to the clusters found in the whole community analysis, except for two of the natural sites (Fig 2b). SIMPER revealed higher abundances of all species observed in the cluster with all CC3 sites, and some CC1, CC2, and natural sites.

Adult community compositions differed significantly among natural sites and CC1, CC2, and CC4, whereas CC3 and natural sites had significantly similar adult community compositions (Fig 2c, pseudo-F(4,15) = 2.095, P = 0.0009). SIMPER revealed that *B*. *capriseus*, *E*. *adescensionis*, *H*. *aurolineatum*, *Parablennius marmoreus*, and *Sphoeroides spengleri* were observed more often over natural sites, whereas *A*. *protabocephalus*, *Mycteroperca phenax*, and *Pareques umbrosus* were seen more often over culvert sites. Adult abundances were significantly higher at natural sites than at CC2 sites (Fig 3a, Tukey’s P = 0.048). Other species indices did not significantly differ among reef categories (Fig 3).

**Fig 4:**
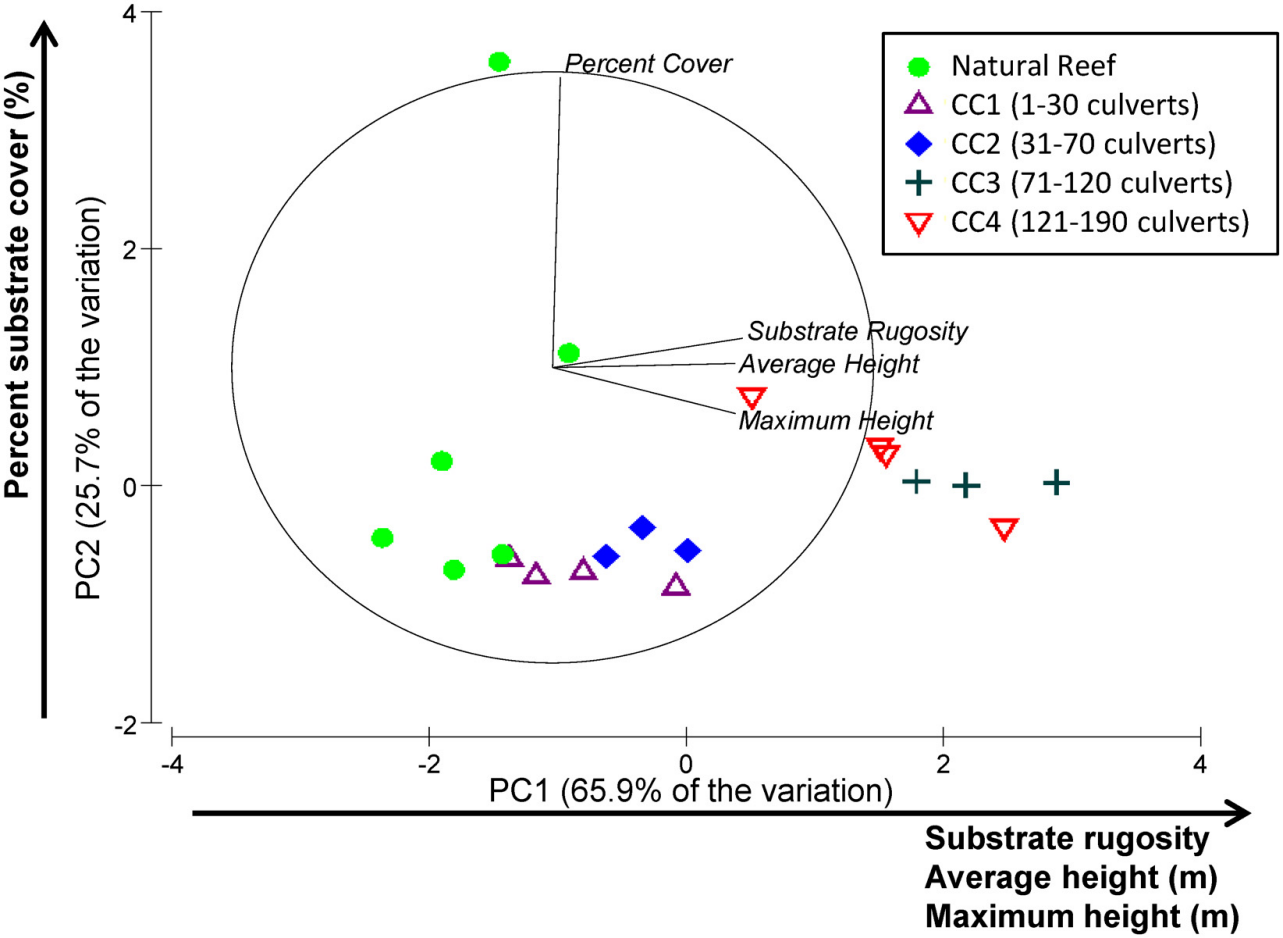
Principal component analysis (PCA) of percent substrate cover, substrate rugosity, average height, and maximum height Vectors point in the direction of increasing values. Distance relative to the outer circle provides indication of how much variation is explained by each vector. If a vector connects to the outer circle, then 100% of the variation in the variable is explained by the two PC axes shown.

PCA analysis produced four PC axes to explain variations in substrate rugosity (1.001–1.288), average vertical relief (0–1.49 m), maximum vertical relief (0–2.95 m), and percent substrate cover (0.76–100%). Two PCs explained 91.6% of variation among the variables tested (Fig 4), and both were interpreted for further analysis. Substrate rugosity, average vertical relief and maximum vertical relief were positively correlated with PC1, while percent cover was positively correlated with PC2 (Fig 4). Natural sites were strongly correlated with PC2, whereas culvert reefs were strongly correlated with PC1. Natural sites had the highest range of percent cover compared to culvert sites. A total of 14 species were repeatedly found at both culvert and natural sites, however no significant correlations were observed between any species and the two PC axes.

### *Lutjanus campechanus* Population and Size Analysis

**Fig 5:**
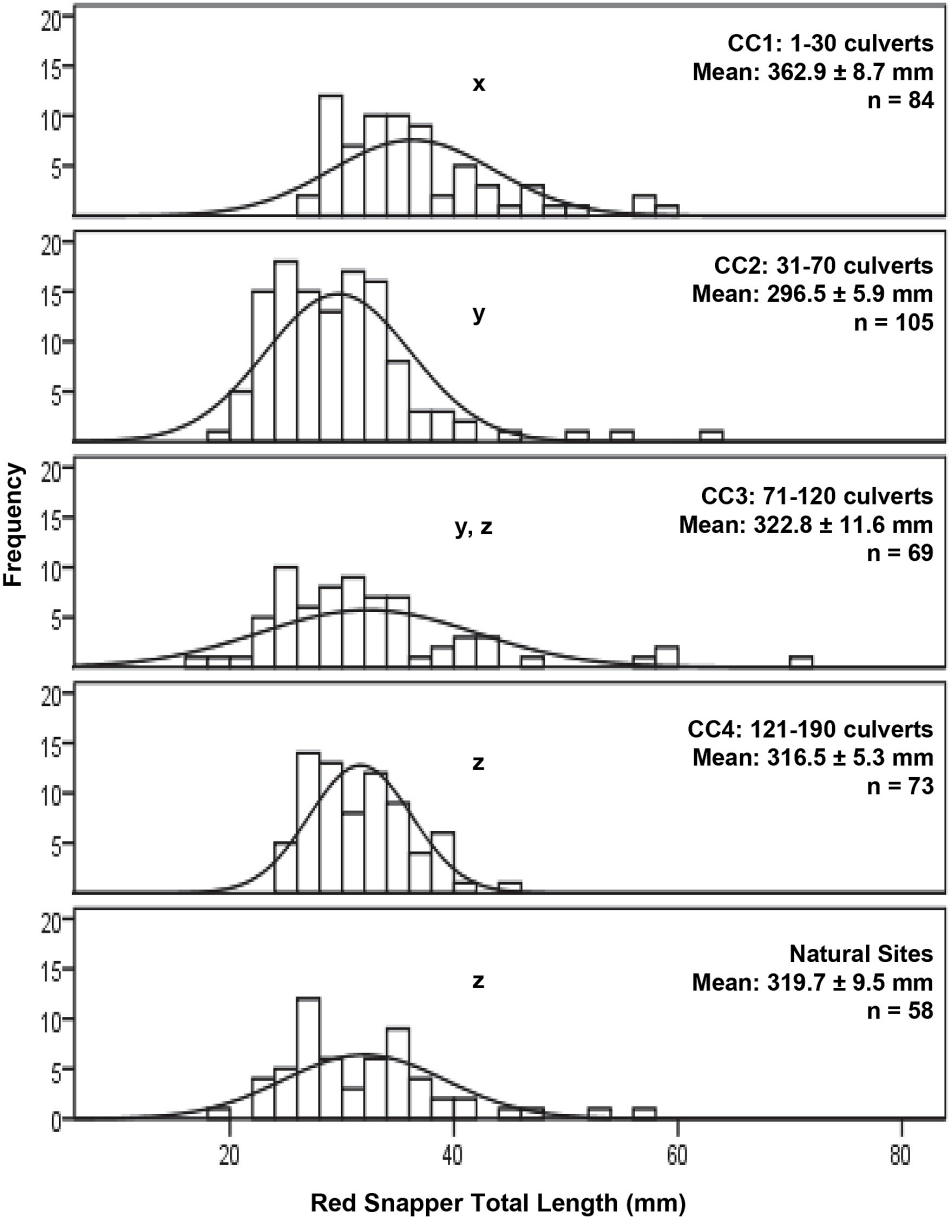
Frequency distributions of *Lutjanus campechanus* total lengths among reef categories in 2014. Non-matching lower case letters indicate reef categories that are significantly different from one another (P > 0.05). Curves represent overall pattern of each frequency distribution.

A total of 732 *L*. *campechanus* were observed in this study. No juveniles were observed at culvert sites, but shoals of juveniles were seen at natural sites. Abundance of *L*. *campechanus* did not significantly differ among any reef categories (F(4,11) = 1.721, P = 0.205), however the highest abundances were observed over natural reefs and CC3 (S2 Appendix). Each site had on average of 41 ± 6 *L*. *campechanus*. Total lengths of *L*. *campechanus* ranged from 127.0 to 710.0 mm, with an mean of 321.0 ± 3.1 mm. Mean total lengths of *L*. *campechanus* were significantly different among reef categories (Welch statistic = 16.733, DF1 = 4, DF2 = 11, P < 0.001). Reefs that had fewer than 31 culverts exhibited significantly higher mean lengths of *L*. *campechanus* than all other reef categories (Fig 5, Games-Howell, P < 0.002). CC2 exhibited significantly lower mean lengths of *L*. *campechanus* than CC4 and natural reefs (Games-Howell, P = 0.006, P = 0.023, respectively). All other reef categories did not significantly differ from one another (Fig 5).

**Fig 6:**
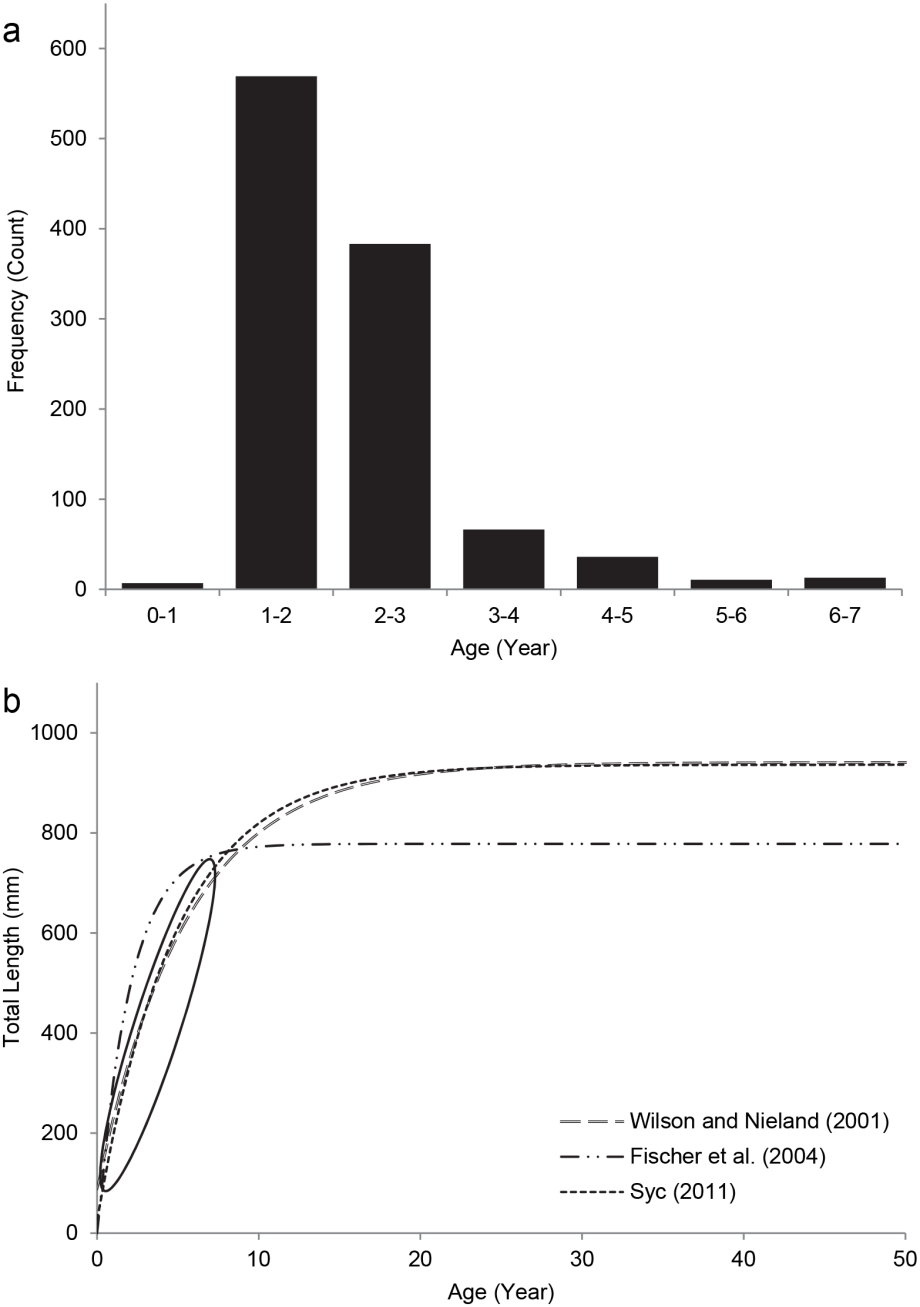
(a) Age distributions of *Lutjanus campechanus* lengths-at-age data from the present study, calculated from the Syc (2011) Von Bertalanffy growth model. (b) Comparison of several growth models for *L*. *campechanus* in the Gulf of Mexico, with the data from the present study indicated by the solid oval.

Using the Syc (2011) Von Bertalanffy growth curve equation coefficients, *L*. *campechanus* age estimates in the present study ranged from 0.6 to 6.8 years old, with an average age of 1.95 ± 0.03 years (Fig 6a). When comparing the predicted age of fish in this study with two other growth models (Fig 6b), average age estimates of *L*. *campechanus* varied by 0.19 years [22,34].

## Discussion

### Reef Type and Complexity

As expected, bare areas surveyed lacked adult and juvenile reef fishes, while areas with artificial reef habitat had diverse populations of reef fishes. Regardless of the number of culverts present in each patch category, reef fish species were observed at culvert reef sites with relatively similar abundances. The lack of difference in fish communities may be due to the structural similarity of the concrete culverts. While culverts found at PS-1047 are not all the same shape and size, the majority are smooth concrete cylinders averaging 1 m wide by 3 m long. A similar study tested the effects of culvert block shape (flat end vs. flush block reefs) and found no significant differences with regard to colonization rate, species richness, or diversity [35].

Some studies suggest that artificial reefs should be structurally complex, and should be similar to natural reefs to better mimic fish habitat [24,36]. However, natural reefs are often more structurally complex than their artificial reef counterparts (i.e. variability in hole sizes), and hold greater species diversity and abundance [36,37]. In the present study, natural reefs were less rugose than artificial reefs, but the side scan sonar method used to assess rugosity could only detect large-scale complexity. Divers in this study noted that natural reefs had a greater variety of benthic invertebrates and more holes. Higher percent substrate cover was also observed at natural reefs, but no statistical differences in community composition, using a multivariate approach, were detected between artificial and natural reefs. A study conducted in the Caribbean Sea also found similar fish community compositions between artificial and natural reefs [24].

In the present study, although natural and artificial reefs had similar fish community compositions, natural reefs and the third culvert category (CC3: 71–120 culverts) had the highest observed species abundance and richness. Assessing adult and juvenile populations may better elucidate observed patterns, because habitat selection is different for both age groups of certain species [38–40]. Juvenile settlement did not explain the higher values at natural reefs and CC3, and may be due to a number of factors, including stochastic processes. However, adults may relocate to other areas, and remain residents for extended periods of time [9,41,42]. Thus, observing more adult individuals in 2013 than in 2014, could be due to other processes, including fishing pressure [43], predation [44], or variation in seasonal temperatures [45,46]. The adult population also seems to be driven by structural density, with highest observed abundances at CC3 and natural sites, and similar community compositions at both categories. This suggests that the intermediate structural density of CC3 may have the optimal density of culverts for reef fish communities.

### *Lutjanus campechanus* Population Analysis

In recent years, populations of *Lutjanus campechanus* have started to recover due to increased management practices and additional habitat [47]. Yet research on populations at artificial reefs continues to be important in order to understand how *L*. *campechanus* are using artificial habitat. In the present study, *L*. *campechanus* abundances did not vary among reef density categories, however highest values were observed at CC3 and natural sites. Although the effect of habitat density on the abundance of *L*. *campechanus* is unclear, shoals of *L*. *campechanus* were observed at all sights studied. Such findings insinuate that PS-1047 holds suitable habitat for this species.

As expected, *L*. *campechanus* juveniles were only seen at low-relief natural reefs, not at culvert reefs. Natural reefs have more complex habitats, with varying substrate sizes, and may indeed serve as nursery grounds for *L*. *campechanus* [3,23]. Predation may also be limited at these low-relief nursery grounds [48]. As adults, *L*. *campechanus* are reported to move to an environment with higher relief [3,23,49], such as the culvert reef at PS-1047. Yet the current study found little variation in the mean lengths of *L*. *campechanus* at natural and culvert reefs. Some young adults might remain at natural patches to continue growing, while others might move to the higher-relief culvert reefs.

Despite similar mean lengths of *L*. *campechanus* at natural and culvert reefs, mean lengths were significantly larger at the lowest culvert density (CC1). This suggests some density dependent effect at the smallest reef patch category, where larger fish could monopolize the structures and drive away smaller individuals. Reports of similar density-dependent behaviors have been observed for *L*. *campechanus* [50] and for another reef-associated fish, *Mycteroperca microlepis* [9].

When lengths were converted into ages, the average age of *L*. *campechanus* in this study was estimated at 2 years old. These age estimates should be verified with otolith samples taken directly from PS-1047 populations, but such a small average age suggests that many *L*. *campechanus* at these patch reefs are not yet sexually mature [3]. Other studies assessed *L*. *campechanus* ages from otolith samples of recreational and commercial catches [22,34,51–53] and fish traps [53], and found much older cohorts than in the present study. Observing such young individuals at PS-1047 suggests that cohorts are aging alongside the young reef [54], or the reef is functioning as a stepping stone for young *L*. *campechanus* to grow before moving to other areas [3].

The findings in the present study suggest that an intermediate density of culverts (71–120 culverts) would be the most efficient use of the material deployed. Moreover, culvert reefs at the intermediate density hold similar species abundances as natural reefs surveyed in this study. In future deployments, an intermediate density of culverts in a 30-m radius will be a more effective and efficient use of concrete structure, and will yield high fish diversity and secondary production.

## Supporting Information

S1 Appendix Presence and absence of species from 2013 and 2014. (DOCX)

S2 Appendix Mean *Lutjanus campechanus* abundances in varying reef categories in 2014. CC1: 1–30 culverts, CC2: 31–70 culverts, CC3: 71–120 culverts, CC4: 121–190 culverts, and Natural: naturally-occurring reefs. Error bars represent standard error (± 1).
(TIF)
