# Supplementary material for: Using Fish Population Metrics to Compare the Effects of Artificial Reef Density
Source: PLoS One. 2015 Sep 30;10(9):e0139444. doi: 10.1371/journal.pone.0139444 (PMC4589336; doi:10.1371/journal.pone.0139444)
Supplement: S1 Appendix — (DOCX) [file pone.0139444.s001.docx]

| **Family** | **Fish Species** | **2013** | | **2014** | | |
| --- | --- | --- | --- | --- | --- | --- |
|  |  | **June** | **July** | **May** | **June** | **July** |
| Apogonidae | *Apogon maculatus^c^* |  | **X** |  | **X** |  |
| Ariidae | *Ariopsis felis^c^* |  |  |  | **X** | **X** |
| Balistidae | *Balistes capriscus^c^* | **X** | **X** | **X** | **X** | **X** |
| Blenniidae | *Hypsoblennius invemar^c^* | **X** |  |  | **X** |  |
|  | *Parablennius marmoreus^c^* | **X** | **X** | **X** | **X** | **X** |
| Carangidae | *Caranx crysos^c^* | **X** | **X** | **X** |  |  |
|  | *Caranx hippos^c^* | **X** |  |  | **X** |  |
|  | *Chaetodipterus faber^c^* | **X** | **X** | **X** | **X** | **X** |
|  | *Decapterus macarellus^c^* | **X** |  |  |  |  |
|  | *Elagatis bipinnulata^a,c^* | **X** |  |  |  |  |
|  | *Selene vomer^c^* |  |  | **X** |  |  |
|  | *Seriola dumerili^b^* |  |  | **X** | **X** | **X** |
|  | *Seriola rivoliana^c^* | **X** | **X** |  | **X** |  |
| Chaetodontidae | *Chaetodon ocellatus^b^* | **X** | **X** |  | **X** | **X** |
|  | *Chaetodon sedentarius^b^* |  |  |  | **X** | **X** |
| Dasyatidae | *Dasyatis americana^a,c^* |  |  | **X** |  |  |
| Gobiidae | *Coryphopterus punctipectophorus^a,c^* |  |  |  | **X** |  |
| Haemulidae | *Anisotremus virginicus^b^* | **X** | **X** |  | **X** | **X** |
|  | *Haemulon aurolineatum^b^* | **X** | **X** | **X** | **X** | **X** |
|  | *Haemulon plumierii^a,c^* |  |  |  | **X** |  |
|  | *Orthopristis chrysoptera^c^* |  |  | **X** |  |  |
| Holocentridae | *Holocentrus adscensionis^a,c^* | **X** |  |  |  |  |
| Labridae | *Bodianus rufus^a,c^* | **X** |  |  |  |  |
|  | *Halichoeres bivittatus^b^* | **X** | **X** | **X** | **X** | **X** |
|  | *Halichoeres burekae^b^* |  |  |  | **X** |  |
| Lutjanidae | *Lutjanus campechanus^b^* | **X** | **X** | **X** | **X** | **X** |
|  | *Lutjanus griseus^c^* | **X** | **X** | **X** | **X** | **X** |
|  | *Lutjanus synagris^c^* |  |  |  | **X** | **X** |
|  | *Ocyurus chrysurus^a,d^* |  | **X** |  |  |  |
| Monacanthidae | *Aluterus scriptus^a,c^* |  |  |  |  | **X** |
|  | *Stephanolepis hispidus^c^* |  | **X** |  | **X** |  |
| Muraenidae | *Gymnothorax moringa^a,c^* |  |  |  |  | **X** |
|  | *Gymnothorax vicinus^a,c^* |  |  |  | **X** |  |
| Ostraciidae | *Lactophrys trigonus^a,d^* |  | **X** |  |  |  |
| Paralichthyidae | *Paralichthys albigutta^a,c^* | **X** |  |  |  |  |
|  | *Paralichthys lethostigma^a,c^* | **X** |  |  |  |  |
| Pomacanthidae | *Holacanthus bermudensis^b^* | **X** |  |  | **X** |  |
|  | *Pomacanthus arcuatus^a,d^* |  |  |  |  | **X** |
|  | *Pomacanthus paru^a,d^* |  | **X** |  |  |  |
| Pomacentridae | *Chromis scotti^c^* |  |  |  | **X** |  |
|  | *Stegastes leucostictus^d^* |  |  |  | X |  |
|  | *Stegastes variabilis^b^* | **X** | **X** | **X** | **X** | **X** |
| Ptereleotridae | *Ptereleotris calliura^a,c^* |  |  |  | X |  |
| Rachycentridae | *Rachycentron canadum^c^* | **X** | **X** |  | **X** |  |
| Sciaenidae | *Pareques umbrosus^b^* | **X** | **X** | **X** | **X** | **X** |
| Scorpaenidae | *Scorpaena plumieri^c^* | **X** | **X** | **X** | **X** | **X** |
| Serranidae | *Epinephelus adscensionis^b^* | **X** | **X** | **X** | **X** | **X** |
|  | *Mycteroperca interstitialis^c^* |  |  | **X** |  |  |
|  | *Mycteroperca microlepis^c^* | **X** | **X** | **X** | **X** | **X** |
|  | *Mycteroperca phenax^c^* | **X** | **X** | **X** | **X** | **X** |
|  | *Rypticus maculatus^c^* | **X** |  | **X** | **X** | **X** |
|  | *Serranus subligarius^c^* | **X** | **X** | **X** | **X** | **X** |
| Sparidae | *Archosargus probatocephalus^c^* | **X** | **X** | **X** | **X** | **X** |
|  | *Diplodus holbrookii^a,c^* | **X** |  |  |  |  |
| Sphyraenidae | *Sphyraena barracuda^a,c^* | **X** |  |  |  |  |
|  | *Sphyraena picudilla^b^* | **X** |  |  |  |  |
| Synodontidae | *Synodus foetens^c^* |  |  |  | **X** | **X** |
| Tetraedontidae | *Canthigaster rostrata^a,c^* |  |  |  | **X** |  |
|  | *Sphoeroides spengleri^c^* | **X** |  | **X** | **X** | **X** |

“X” indicates presence of the species. Numbers indicate the following about the species:

^a^ = only one or two individuals observed, ^b^ = adults and juveniles observed,

^c^ = only adults, ^d^ = only juveniles
